# The spatial distribution of colorectal cancer relative risk in Iran: a nationwide spatial study 

**Published:** 2020

**Authors:** Mohamad Amin Pourhoseingholi, Hadis Najafimehr, Amir Kavousi, Leila Pasharavesh, Binazir Khanabadi

**Affiliations:** 1 *Basic and Molecular Epidemiology of Gastrointestinal Disorders Research Center, Research Institute for Gastroenterology and Liver Diseases, Shahid Beheshti University of Medical Sciences, Tehran, Iran *; 2 *Gastroenterology and Liver Diseases Research Center, Research Institute for Gastroenterology and Liver Diseases, Shahid Beheshti University of Medical Sciences, Tehran, Iran*; 3 *Department of Epidemiology, School of Public Health and Safety, Shahid Beheshti University of Medical Sciences, Tehran, Iran *

**Keywords:** Colorectal Cancer, Relative risk, Bayesian model, Poisson regression, Spatial analysis

## Abstract

**Aim:**

The aim of this study was to estimate the standardized incidence rate (SIR) and also the relative risk (RR) of colorectal cancer (CRC) in Iran and to determine the distribution of CRC risk in a map after adjusting socioeconomic risk factors.

**Background:**

The growth of CRC incidence rate in Iran is a major public health problem and identifying high-risk regions is essential for further intervention.

**Methods:**

For this cross-sectional study, all CRC cases that occurred in 30 Iranian provinces between 2005 and 2008 were collected according to the International Classification of Diseases (ICD-10). In addition, socioeconomic information was extracted from statistical center of Iran. Bayesian and Poison regression models were fitted to identify significant covariates. For RR estimating, the spatial analysis using GIS technique was carried out.

**Results:**

The Bayesian method with increasing precision of the parameter estimates had a better fit. According to spatial model, East Azerbaijan province had a high (11.14) and South Khorasan province had a low (0.22) risk of CRC in the period of study. SIR for the male population was 1.92 ± 3.25, and for the female population it was 1.85 ± 3.37.

**Conclusion:**

There is a non-uniform spatial pattern of CRC risk in Iran. According to the results, North, Northwest and some parts of West and Central provinces of Iran are identified as high-risk areas; thus, it is recommended that health policymakers, especially in these areas, have more intervention measures. Further studies are needed to map the RR adjusted for nutrition factors.

## Introduction

 Based on official reports, nearly 20 million people in the world are affected by cancer, and according to time-trend analyses, this number would increase to more than 30 million in 2020. Additionally, the number of new cases in 2030 may reach to 21.7 million people (1). Referring to reports of the Iranian Ministry of Health and Medical Education (MOHME) and also some other studies on cancer epidemiology, the most common cancers in Iran are stomach, esophagus, breast, prostate and colon and rectum (colorectal) (2, 3). The colorectal cancer (CRC) with 400,000 annual deaths in the world is the third cause of cancer death (4). Based on the reports of World Health Organization (WHO), the CRC incidence rate has rapidly increased in the past decades in Eastern Europe and Asia, including Iran, particularly for men (5). 

In the pathogenesis of CRC, individual lifestyle and personal habits such as smoking, alcohol consumption, diet and physical activities are known to be related with the risk of disease (6, 7). Recent studies revealed that there are substantial differences in disease risk and morbidity rate across regions (8-11). Therefore, identification of areas with the accumulation of cases for health policy makers is of prime importance (12). The spatial analysis techniques by using Geographic Information Systems (GIS) may be useful in measuring the distribution of the disease and determining high-risk areas and related environmental factors (13, 14). GIS, widely used in epidemiological researches, allows visualizing, exploring, and modeling of health patterns via mapping the distribution of health outcomes (15). 

There are few studies on the Relative Risks (RRs) of CRC in Iran that cover all provinces, and none of them investigate the spatial distribution of the disease (16). The present study provides an estimate of the RRs of CRC at the nation-wide level and a geographical distribution of the risks and also evaluates the association of socioeconomic factors with the risk of CRC. 

## Methods

**Data collection**

The data for our cross-sectional study were provided by the Iranian Ministry of Health and Medical Education (Iran Cancer Registry Report). All cases from hospital records and diagnosis of CRC according to the International Classification of Diseases (ICD-10: C19 Diagnostic Code) were collected from 30 provinces in Iran from January 2005 to December 2008 (17). The reference population by gender, information about the average household income/106 Rials (MHI) and unemployment rate (UER) for each province was extracted from the 2006 census by the Statistical Center of Iran (SCI) (18). The SCI Population and Housing Census have been nationally implemented since 1956; before 2008 it was carried out every 10 years and every 5 years thereafter. During the study period, the population at risk was almost stable, so we used the 2006 census Population and Housing. 

**Statistical analysis**

In our study, the Standardize Incidence Rate (SIR) was calculated for all provinces for each gender. The SIR for each province was obtained from the ratio of the observed and expected number of cases in that province. The expected number of cases was calculated with the aid of internal standardized method. The SIR is considered as crude estimate of relative risk (RR), so its smoothed estimates was obtained with the aid of Generalized linear models (GLM) as follows: A Poisson distribution with $E_{i}\theta_{i}$ mean was considered for the number of cases in province i, where $E_{i}$ represents the expected incidence and $\theta_{i}$ represents the RR in province *i*. Thus, in order to fit the model to data, the GLM with a logarithm (log) link function was used. We provided two statistical methods based on the GLM: non-spatial model and spatial model with random effect components by considering auto correlation structure in data. The random effect model was specified as:

$\mathrm{Log}\left(\theta_{i}\right)=\log\left(E_{i}\right)+\alpha +\beta_{1}{\mathrm{UER}}_{i}+\beta_{2}{\mathrm{MHI}}_{i}+u_{i}+\delta_{i};i=1.\ldots \mathrm{.n}$

Where α is the intercept; u and δ random effect components are the non-spatial and the spatial terms, respectively; UER and UER are the covariates for model adjusting; and $\beta_{1}$ and $\beta_{2}$ were regression coefficients describing associations with the UER and MHI, respectively. In the spatial method for estimating the model parameters, the Bayesian model was adopted using prior distributions. The prior distribution are as follows: Conditional Auto Regressive (CAR) model for spatial term in order to enter adjacent effects, Normal (0,1) for covariates, Normal (0,100) for α, and Normal (0, $\sigma^{2}$) where σ~ Normal (0, 1000) for non- spatial term (19, 20). We utilized the Marcov Chain Monte Carlo (MCMC) simulation technique by 50000 iterative samples. The convergence was achieved after 2000 iterative burn-in. For the analyses of data in spatial method, the WINBUGS program was used (21). For non- spatial method the same adjusted model was employed without considering random effect components and the data was analyzed by SPSS 21 software. The Deviance information criteria (DIC) and -2log likelihood were used for model comparison. All maps were created by ArcGIS10. 

## Results

According to the Iranian Ministry of Health and Medical Education reports, a total of 21,543 CRC cases were found between 2005 and 2008 in Iran, including 56.42% male and 43.58% female. The number of observed and expected CRC cases in each province is presented in table 1.

**Table 1:** Observed and Expected cases of Colorectal Cancer in Iranian Provinces: 2005-2008

Province	Observed		Expected	
	Female	Male	Female	Male
E- Azerbaijan	376	476	32.96	43.87
W- Azerbaijan	237	302	330.51	63.80
Ardabil	2028	2612	192.51	251.02
Esfahan	590	799	700.90	949.62
Ilam	27	48	84.22	113.27
Bushehr	64	85	131.81	190.32
Tehran	2088	2586	2055.28	2806.18
Chaharmahal	82	120	134.35	175.52
S-Khorasan	23	27	99.05	130.99
R-Khorasan	654	845	877.34	1142.36
N-Khorasan	37	41	129.13	163.41
Khuzestan	370	483	33.52	45.71
Zanjan	69	116	152.20	195.87
Semnan	79	109	90.55	122.98
Sistan	58	79	373.32	496.58
Fars	391	568	671.95	896.54
Qazvin	109	130	176.29	237.41
Qom	86	151	160.68	218.32
Kordistan	110	155	223.91	296.72
Kerman	169	176	409.26	550.51
Kermanshah	218	252	27.99	36.39
Kohgiluyeh	38	47	98.75	130.52
Golestan	154	200	256.21	326.99
Gilan	358	528	380.20	487.34
Lorestan	125	168	265.64	355.26
Mazandaran	407	490	458.75	596.46
Markazi	112	141	210.82	277.46
Hormozgan	64	102	213.84	294.88
Hamadan	136	182	266.77	348.41
Yazd	129	137	149.29	210.28

**Table 2:** Summary of Colorectal Cancer incidence by sex in Iran: 2005-2008

			SIR^*^			
	No. of cases	Incidence rate (per 10^5 ^person-years)	Mean	SD^**^	Max	Min
Sex						
Male	12155	10.16	1.92	3.25	10.85	0.16
Female	9388	7.88	1.85	3.37	11.41	0.16
Both sexes	21543	9.02	1.83	3.28	11.11	0.16

*Standardize incidence rate; **Standard deviation

**Table 3:** Parameter estimates and model comparison in spatial and non- spatial adjusted models for the risk of Colorectal Cancer

	Spatial model	Non- spatial model
	Exp(β) (95% CI)	Exp(β) (95% CI)
Factor		
UER^*^	1.04 (0.94,1.15)	1.13 (1.02,1.43)
MHI^**^	1.01 (0.99,1.03)	1.46 (1.06,1.57)
Model comparison criteria		
DIC^***^	291.82	19028.86
-2log likelihood	231.63	19018.65

*Unemployment rate; **Mean household income; ***Deviance information criteria

**Figure 1:**
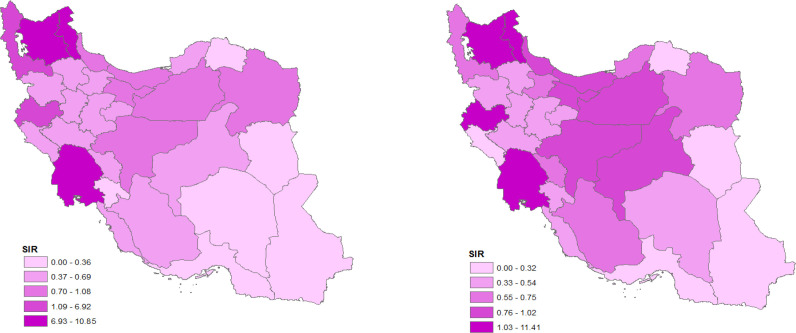
Spatial pattern of colorectal cancer’s standardized incidence rate (SIR) in Iranian male (a) and female (b): 2005-2008

In the male population, the SIR (with mean of 1.92) was higher than females (with mean of 1.85). The incidence rate index for males (10.16) was higher than the female population (7.88). Table 2 shows a summary of incidence by sex in the whole provinces. For the comparison between male and female population, the maps of the SIR by sex are provided in figure 1. According to figure 1, the North and some parts of Western regions had high CRC incidence rate for men while, Central provinces had a higher rate for women. 

**Figure 2:**
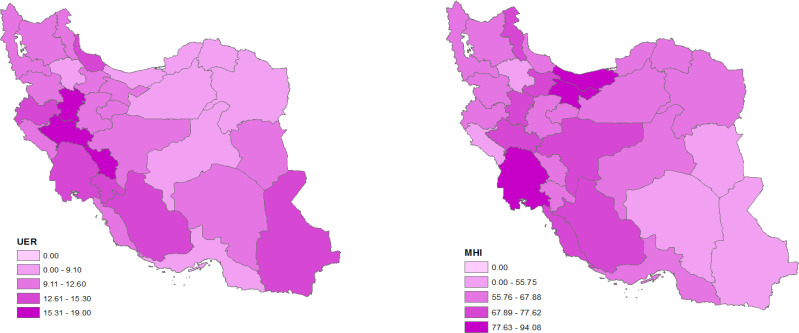
Spatial pattern of unemployment rate (UER) (a) and mean hosehold income (MHI) (b) in Iran

In the present study for RR estimating in adjusted models, we used UER (mean ± SD: 11.64% ± 3.18%) and MHI (mean ± SD: 66.46Rials ± 12.04Rials) covariates. The maximum UER (19%) belongs to Lorestan province and the minimum (6.40) belongs to North Khorasan province; the maximum MHI (94.08) belongs to Mazandaran province and minimum (33.22) to Ilam province. The pattern of covariates is presented in figure 2. The results of the regression coefficients estimate in spatial and non-spatial models are illustrated in table 3. However, an increase in both covariates resulted in higher risk of CRC, and posterior estimates in the spatial model showed that the covariates was not significant. Comparison between spatial and non-spatial models with the aid of goodness of fit criteria showed that spatial model had a better fit to the observed data (Table 3). Based on the RR estimates, East Azerbaijan province had the highest RR (11.14) and South Khorasan province had the lowest (0.22). 

**Figure 3:**
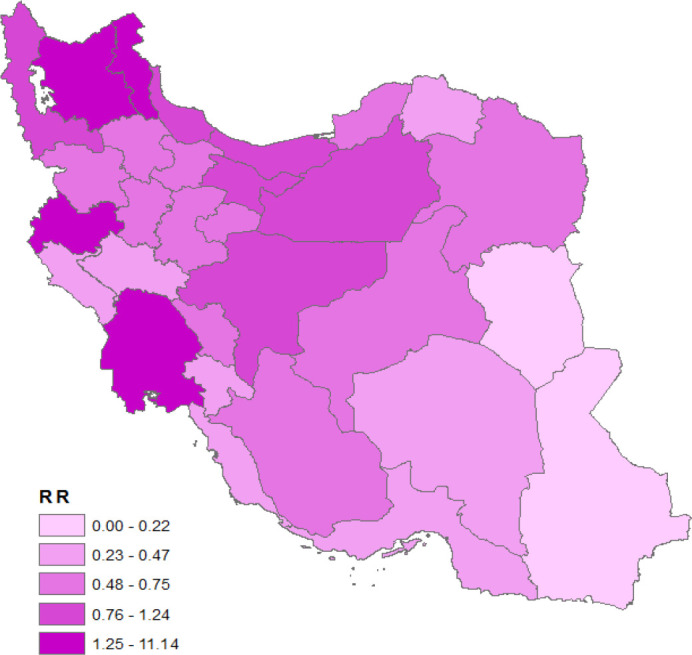
Spatial distribution of colorectal cancer’s relative risk (RR) in Iran: 2005-2008

The pattern of spatial distribution of CRC RR is prepared in figure 3. According to the map, it is clear that the Northern, North Western, some parts of West and central provinces are considered as high-risk regions. 

## Discussion

Studies imply that the incidence of CRC is increasing over time, but there are regional differences (22). These differences could be explained considering individual and societal discrepency in economy, ethnicity, dietary and lifestyles in different geographical regions. Based on previous systematic review on CRC between 2003 and 2014 in Iran, in addition to increasing CRCs throughout the country, the prevalence varies from region to region (23). Moreover, the difference in the distribution of CRC across the country has been confirmed by Pakzad et al. study that used the hot spot (Getis-Ord Gi) method, and identified the North and Central regions of Iran as the highest incidence of CRC in men compared to women (24). Mahaki et al. using the shared component model, introduced the North, Centeral and Northwest regions as high-risk areas (25). 

The main objective of present study with the aid of investigating the CRC geographical distribution, is the identification of high-risk Iranian provinces. The results of this study are consistent with Iranian studies on CRC. Regarding the province distribution of relative risk of CRC, we found similar results by detecting clustering in Central and Northwestern regions. Among the area-level factors that predict the health status of population, the level of education, socioeconomic status and poverty are major factors in health inequalities (26). The Fukuda et al. study in Japan, in terms of geographical differences in the incidence of CRC, reported urban mortality, unemployment rate, individual income, and educational level as risk factors associated with CRC mortality rates (27). Carroll et al. modeled the CRC incidence at county level in Georgia and found that median household income and percentage of persons below poverty level acted as predictors of CRC in Northern and Southern counties, respectively (28). In our study, we evaluated the impact of two socioeconomic, UER and MHI factors. In the non-spatial model, the selected factors had a significant association with the CRC risk, while it was more fitted in the spatial model and these factors did not have a significant relationship. The spatial approach, using the regional impacts in the Bayesian method, increased the accuracy of estimates in our regional-level data and was selected as a better model based on appropriate criteria.

 The previous study on the relationship between CRC risk and dietary habit suggests that the excessive consumption of red meat and low fiber intake can play an important role in the incidence of CRC (29). Besides, it is clear that income can affect food habits, and foods containing red meat and fast foods are more expensive than vegetable foods in Iran. The maps of socioeconomic factors that were evaluated in this study revealed that the people in North and West part of Iran have a higher level of welfare. Consequently, the distribution map of RR in our study showed that these regions were at higher risk of CRC.

Due to limitation in availability in province-level information in Iran, we only employed the UER and MHI information but there may be other variables to be verified. Notably, in some provinces in Iran there is misclassification problem in tt registered data because of poor quality in data registering which may alter results. The misclassification error occurs when people travel to the prosperous provinces for better midical care and their data is incorrectly registered at the prosperous province (30, 31). This type of error may results imprecision in the adjacent effects estimation and leading to nonsignificant results (32, 33). Another limitation of the study was the lack of up-to-date data due to the absence of free access to national data on cancer registration in Iran. Access to such data was only available until 2008. The strength of this study was the use of powerful statistical methods in parameter estimation through Bayesian method. In addition, the use of spatial analysis was important in this study, because of its computational power, which has rerely been conducted in Iran. 

 In conclusion, knowing area-level information and spatial distribution of the risk of diseases can inform the planning of health care services. According to our findings, the North, Northwest and some parts of West and Central provinces of Iran are identified as high-risk areas for CRC. Our findings can help to inform health policy makers and implement interventions to reduce the risk of CRC in high-risk regions. Further studies are needed to identify other factors such as diet influencing the CRC risk at provincial and regional level.
